# The impact of health insurance programs for children: evidence from Vietnam

**DOI:** 10.1186/s13561-016-0111-9

**Published:** 2016-08-05

**Authors:** Cuong Nguyen

**Affiliations:** National Economics University, Hanoi, Vietnam

**Keywords:** Child health insurance, Impact evaluation, Health care utilization, Out-of-pocket expenditures, Vietnam, I10, G22, H43

## Abstract

This study assesses the impact of children’s health insurance programs on health care utilization and health care expenditures of children from 6 to 14 years old in Vietnam using four rounds of the Vietnam Household Living Standard Surveys from 2006 to 2012. We find a positive effect of both student and free health insurance programs on the number of health care visits. This positive impact tends to increase over time, and the impact of the free health insurance program is larger than the impact of the student health insurance program. Regarding out-of-pocket health expenditures per visit, we find a reducing effect on this outcome of the free health insurance program but not the student health insurance program.

## Background

Child health has received a great deal of attention in all the countries. Improvement of child health in low-income countries is challenging because of nutrition problems and poor health care services. The rate of stunting among children under-five years of age (height-for-age below −2 SD) was 36 in Africa and 27 % in Asia in 2011 [1]. In low-income countries, the under-five mortality rate was 76 deaths per 1000 live births in 2013, while the under-five mortality rate in high-income countries was just around 6 deaths per 1000 live births [2]. Improving child health and reducing the mortality rate are among important objectives of Millennium Development Goals.

Among health programs, health insurance is a key one to help households improve health and avoid catastrophic health expenditures. Firstly, health care costs are often high for the poor households, and this high cost may lead to a delay in using health care when they are sick. Thus, health insurance can increase health care utilization, and improve health status of people. Secondly, when a person suffers from negative health events such as accidents or chronic diseases, their medical expenses increase substantially (for example, [3–5]). To cover the health care costs, poor households might have to reduce other consumptions and investment, and sell some of their goods. There are evidences that even a short term health event can push some households into long-term poverty (e.g., [6, 7]). Health insurance helps households reduce the out-of-pocket expenditures including catastrophic health expenditures (e.g., [8–10]).

Vietnam has been very successful in poverty reduction in the recent decades. However, child health remains an important problem in Vietnam. In 2011, 23.3 of children under five in Vietnam were under normal height (height-for-age below −2 SD) and 12 % of children under five years old were under normal weight (weight-for-age below –2SD). Children in low expenditure quintiles are more likely to be under normal weight and height compared to those in high expenditure quintile [11]. Children in poor household are also more vulnerable to illness [11]. Without proper treatment, illness can have adverse impacts on children’s health and education. These adverse impacts can be mitigated if children have health insurance.

The government of Vietnam has implemented policies to increase the health insurance coverage for children. Children under 6 years old are provided with free health care services. For children from 6 to 14 years old, there are two main health insurance programs, which are operated by Vietnam Health Insurance Organization (VHI) on non-profit and public basis. The first is student health insurance, which is provided for school children on a voluntary basis. The second program is free health insurance for the poor children, and children in ethnic minorities and other policy households such as households with war merit or invalid members. According to Vietnam Household Living Standard Surveys, 62.8 of children age from 6 to 14 were enrolled in at least a health insurance program in 2006 and this rate increased to 88.6 % in 2012.

Although there is no doubt about the necessity of health insurance programs in Vietnam, there exist questions on their effectiveness. They are sometimes to blame for poor health care services (e.g., [11, 12]). Insured people must use the health care services in a local medical clinic that they registered when purchasing health insurance. The mechanism can limit health care utilization of people, especially those with high mobility. In addition, there is a large geographic variation in the quality of health care services in Vietnam. In mountainous and remote areas, health care services are poorly provided [13]. For example, the average distance from households under the most difficult communes (under the Program 135) in Vietnam to the nearest hospital is around 21 km [13, 14]. On the contrary, in urban areas, especially in large cities, hospitals are often overwhelmed by high utilization. As a result, 30 % of insured people did not use health insurance when using health care services in 2012 (according to the Vietnam Household Living Standard Survey 2012). People tend to chose to pay directly to health service providers to receive fast health treatment.

The impact of health insurance has been receiving a great amount of attention from scientists as well as policy makers. There are a large number of empirical studies on the impact of health insurance programs in both high and low income countries. There are two important issues in empirical studies. Firstly, the impact of health insurance on health care utilization and out-of-pocket expenditures is not known a priori. Positive impacts of health insurance on health care demand and utilization are found in several studies such as [15–19]. However, other studies find limited effects of health insurance on health care utilization and expenditures e.g., [20–22]. Regarding the effect on out-of-pocket expenditures, several studies find a significant effect of health insurance on out-of-pocket expenditure reduction e.g. [8–10], while others do not find such a significant effect (e.g., [5]). Secondly, most studies look at the impact of health insurance on overall population, not children, e.g., [10, 15–19, 21, 22]. Child-targeted health insurance programs have been introduced in few low and middle income countries, and there is little evidence on the impact of these health insurance programs [23].

In Vietnam, the impact of health insurance has been evaluated quantitatively in a number of studies, and the empirical results are not consistent. Most studies find that health insurance helps the insured people increase health care utilization [8, 24–27]. Health insurance is also found to reduce out-of-pocket health expenditures [8, 24–26]. On the contrary, [28] does not find a significant impact of health insurance on health care utilization, and [27] does not find a significant impact on out-of-pocket health expenditures.

Regarding child health insurance, two studies assessing the impact of free health insurance on children under age 6 in Vietnam are [23] and [29]. Nguyen et al. [29] finds a positive impact of health insurance on hospital visits of children and a reduction in health expenses, whilst [23] show a positive impact of health insurance on health care visits of children under age 6, but no significant impacts on expenditures per visit. Possible reasons for different findings in these two studies are differences in data sets and methodology. Nguyen et al. [29] uses data from the 2004 and 2006 Vietnam Living Standard Surveys (VHLSS) and difference-in-differences estimators, whilst [23] relies on data from the 2006, 2008 and 2010 VHLSSs, and regression discontinuity regressions^1^.

Empirical findings on the impact of health insurance on children’s health care, especially in Vietnam, remain limited. Thus in this study, we will examine whether the student health insurance and free health insurance affect health care utilization and health spending of children from 6 to 14 year old in Vietnam. This study is expected to have several contributions. Firstly, we provide empirical findings on the impact of health insurance on children aged from 6 to 14. This group of children have not been assessed in previous studies. The impact of health insurance on children can be different from the impact of health insurance on adults. Moreover, the decision to use health care service does depend not only on children but also heavily on their parents and caregivers. Secondly, the study will measure the effect of two health insurance programs for children including student health insurance and free health insurance. The two types of health insurance are targeted at different groups and they can have different impacts on children. Thirdly, we will use more recent data from Vietnam Household Living Standard Surveys from 2006 to 2012. Health insurance as well as health care have been change significantly in Vietnam, and the impact of health insurance can differ from one to another period.

The paper is structured into four sections. The second section presents data and methods used in this study. This section also describes health insurance programs and health care utilization of children in Vietnam. The third section discusses empirical results of the impact measurement of health insurance programs on children. Finally, the fourth section presents some discussions and conclusions.

## Method

### Data set

In this study, we will use data from the recent Vietnam Household Living Standard Surveys (VHLSS) in years 2006, 2008, 2010 and 2012. These surveys were conducted by General Statistical Office of Vietnam with technical supports of the World Bank. The sampling frame of the 2006 and 2008 VHLSSs is based on the 1999 Population and Housing Census of Vietnam. Since 2010, VHLSSs use the 2009 Population and Housing Census of Vietnam as the sampling frame. Each of the 2006 VHLSS and the 2008 VHLSS covers 9,189 households, while each of the 2010 VHLSS and the 2012 VHLSS covers 9,399 households. The VHLSSs are representative for the national, rural and urban, and regional levels.

It’s very useful that VHLSSs have a sub-sample of panel households and individuals. In the VHLSSs, communes are selected randomly as primary sampling units. The number of selected communes in each of the 2006 and 2008 VHLSSs is 3,063, while the number of selected communes in each of the 2010 and 2012 VHLSSs is 3,132.^2^ Around three households are randomly selected in each commune. Regarding panel data, in each VHLSS, 50 % of communes are randomly selected and all the sampled households in these selected communes will be resampled in the succeeding VHLSS to construct panel data. The 2006 and 2008 VHLSSs contain a panel of 4,090 households and 15,475 individuals, while the 2010 and 2012 VHLSSs contain a panel of 4,157 households and 15,011 individuals. There are no panel data between the 2008 VHLSS and the 2010 VHLSS, since they rely on different sampling frames. The attrition rate in the panel data of VHLSS is around 8 % mainly because of migration problem. However, households in the panel data are still representative and there are no serious attrition problems in VHLSSs [30].

The VHLSSs contain very detailed information on household and individual characteristics. Information on households and individuals includes demography, employment and labor force participation, education, health, income, expenditure, housing, fixed assets and durable goods, participation of households in poverty alleviation programs. The surveys contain information on enrolment in different health insurance types, out-of-pocket expenditures on inpatient and outpatient treatments, other expenses on health care, health care utilization, the number of health care visits during the 12 months before the interview for all the sampled individuals.

### Health insurance and health care of children in Vietnam

In Vietnam, health insurance has been implemented by the government since 1992. Nowadays, there are three main schemes of health insurance in Vietnam. The first is compulsory health insurance which is applied for employees in formal sectors.^3^ The second is voluntary health insurance. The third is free health insurance, which is provided freely for the poor and people in policy families such as families with war merit and invalid members and ethnic minority families. The compulsory and free health insurance are provided by the government, while the voluntary health insurance can be provided by both public and private health insurance providers.

**Fig. 1:**
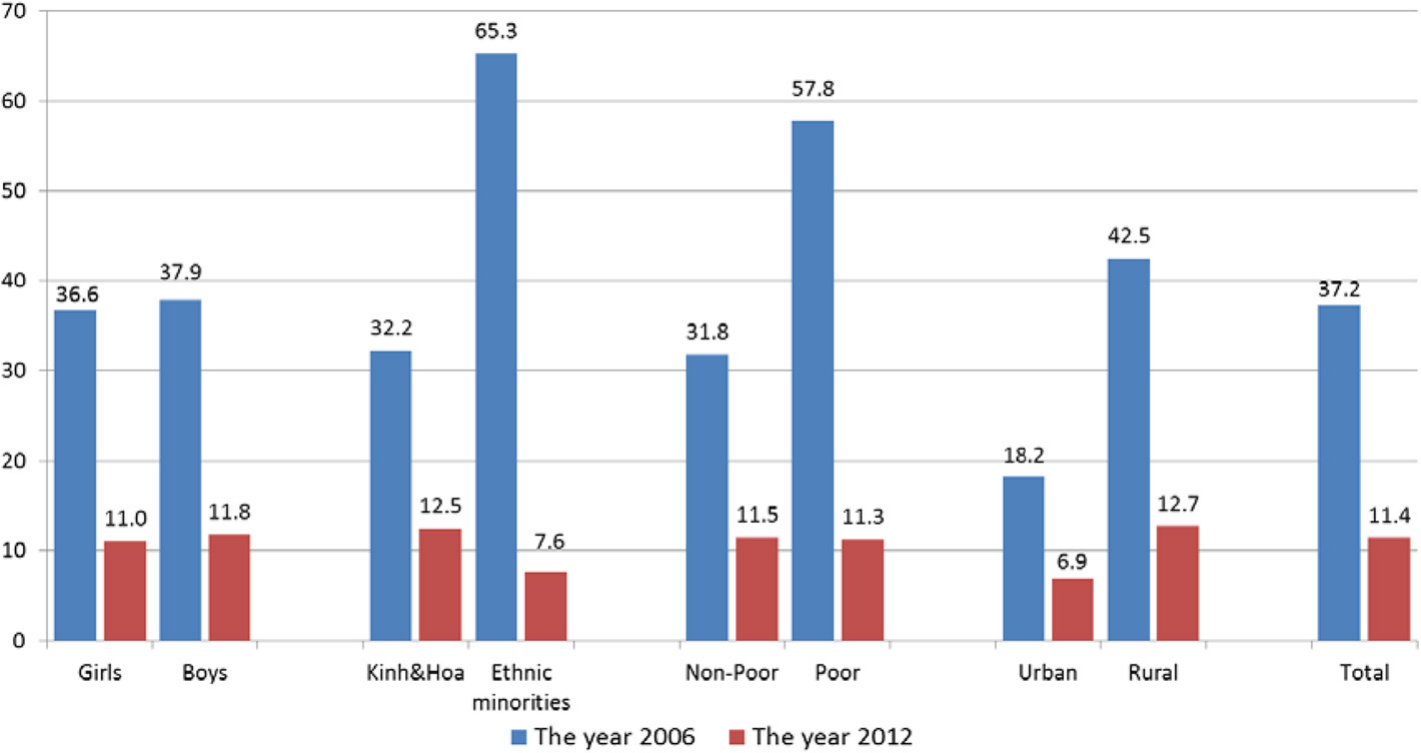
Percentage of children without a health insurance. Source: Estimates from VHLSSs 2006 and 2012

Health insurance for children have been increasing during the recent years. The percentage of children from 6 to 14 years old without health insurance decreased from 37.2 % in 2006 to 11.4 % in 2012 (Fig. 1). Rural children and Kinh/Hoa children are less likely to have a health insurance than urban and ethnic minority children, but this gap is not large. In the recent years, children from ethnic minorities are provided with free health insurance from the government.

Children under age 6 are provided with free health insurance. The main programs of health insurance for children since age 6 are student health insurance and free health insurance. The student health insurance program is voluntary, and users must pay for that. The average fee of student health insurance is around VND 80,000 (approximately USD 4 in January 2012) for one year. It should be noted that the schooling rate is very high in Vietnam, at around 95% for the primary and lower-secondary school. Children who are not enrolled in school can purchase voluntary health insurance. The proportion of children having this kind of health insurance is very low, around 1 % in the 2012 VHLSS. In this study, we define children with student health insurance as those who have either student health insurance or other types of voluntary health insurance^4^.

The second program of health insurance is free health insurance for the poor, ethnic minorities in difficult areas, and policy families. The provision of health insurance for the poor has been supported by “Health Care Fund for the Poor” (HCFP) since 2003. The annual amount that is used to subsidize a beneficiary is about VND 70000 (approximately USD 3.5) for one year. Members in households who are classified as the poor by commune authorities can be eligible for this health insurance program. In addition, children in policy families (including families with war merit and invalid members, and families in ethnic minorities, and areas of special difficulties) can be also provided with free health insurance.

Table 1 presents the coverage of the two health insurance programs for different child groups over the period 2006–2012. The two health insurance programs are mutually exclusive. The coverage of the both health insurance programs increased overtime. The percentage of children who were insured with the student health insurance increased from 52.3 % in 2006 to 62.8 % in 2012. During the same period, the percentage of children insured with the free health insurance increased from 10.4 % to 25.7 %. It should be noted that there was a shift from student health insurance to free health insurance for ethnic minority and poor children due to the government’s expansion of free health insurance. The free health insurance program has reached the poor and ethnic minority children very well. In 2012, 80 of ethnic minority children and 68 % of poor children were enrolled in the free health insurance program.

**Table 1:** Percentage of children with student health insurance and free health insurance

	Percentage of children having student health insurance				Percentage of children having free health insurance			
	2006	2008	2010	2012	2006	2008	2010	2012
By gender								
Girls	52.5	64.0	58.9	63.4	10.9	18.3	23.5	25.5
Boys	52.2	62.3	59.5	62.3	10.0	19.6	22.3	25.9
Ethnicity								
Kinh, Hoa	59.6	67.1	71.8	76.5	8.3	14.3	8.9	11.0
Ethnic minorities	12.3	42.9	10.5	12.3	22.4	42.7	77.3	80.1
Poverty status								
Non-Poor	61.8	70.5	72.7	76.4	6.5	11.4	8.5	12.1
Poor	17.1	33.0	29.9	20.5	25.2	49.8	54.2	68.2
Urban status								
Urban	76.6	76.7	83.0	84.2	5.2	7.3	5.9	8.8
Rural	45.6	59.5	52.3	56.7	11.9	22.1	27.8	30.6
Total	52.3	63.2	59.2	62.8	10.4	18.9	22.9	25.7

Source: Estimates from VHLSSs 2006, 2008, 2010 and 2012

There might be at least two possible reasons why some school children do not have health insurance. Firstly, health insurance fees can be relatively costly for some poor households. According to a survey on willingness to pay for voluntary health insurance which was conducted by National Economics University and World Bank in 2005, 20% of people did not buy health insurance because of the high cost [31]. Secondly, as mentioned in the introduction section health insurance is sometimes to blame for poor health care services and complicated payment request. Some households are not interested in having health insurance for their children (National Economics University and World Bank: Results from willingness to pay for health insurance survey, unpublished).

To understand selection into child health insurance, we run a multinomial logit regression of health insurance of different explanatory variables using the sample of children aged 6 to 14 in the 2006, 2008, 2010 and 2012 VHLSSs. Children are enrolled in either student health insurance or free health insurance. The reference group is children without any health insurance. The description and summary statistics of explanatory variables are presented in Table 6 in Appendix. Since the 2006 and 2008 VHLSSs are based on a different sampling frame from the 2010 and 2012 VHLSS, we run one model using the pooled 2006 and 2008 VHLSSs and another model using the pooled 2010 and 2012 VHLSSs.

Table 2 shows children from Kinh and Hoa, and those from high income households are more likely to be enrolled in the student health insurance program but less likely to be enrolled in the free health insurance program. Schooling children are strongly correlated with student health insurances as well as free health insurance. Children in high income households are more likely to have student health insurance. Children in low income households are more likely to have free health insurance, since the free health insurance program is targeted at the poor and disadvantageous groups. Education of household heads and spouses are strongly correlated with student health insurance. Regarding the free health insurance, education of household heads and heads’ spouse is less correlated with free health insurance of children. However, children in a household with a head or head’s spouse with post-secondary education are more likely to have free health insurance. It implies the role of parental education in getting children insured by health insurance.

**Table 2:** Multinomial logit regression of the probability of children having health insurance

Explanatory variables	Estimation using VHLSSs 2006 and 2008		Estimation using VHLSSs 2010 and 2012	
	Enrolled in student health insurance in 2008 (yes = 1, no = 0)	Enrolled in free health insurance in 2008 (yes = 1, no = 0)	Enrolled in student health insurance in 2008 (yes = 1, no = 0)	Enrolled in free health insurance in 2008 (yes = 1, no = 0)
Age	0.0122	−0.0897***	−0.0407*	−0.0052
	(0.0178)	(0.0258)	(0.0241)	(0.0285)
Sex (male = 1, female = 0)	−0.0250	0.0521	−0.0457	−0.0977
	(0.0757)	(0.1061)	(0.1056)	(0.1313)
Ethnic minorities (yes = 1)	−0.5868***	0.0549	−0.6287***	2.0530***
	(0.1190)	(0.1532)	(0.1811)	(0.1689)
Attending school	2.4796***	1.2287***	2.4920***	0.8578***
	(0.1744)	(0.1974)	(0.2478)	(0.2255)
Log of per capita income	0.4629***	−1.3994***	0.6031***	−1.4203***
	(0.0797)	(0.1233)	(0.1034)	(0.1444)
Household size	−0.0125	0.0060	0.1177***	0.0051
	(0.0268)	(0.0376)	(0.0395)	(0.0446)
Proportion of children in households	0.4780	1.0777***	0.1684	−0.5964
	(0.2989)	(0.3987)	(0.4015)	(0.4757)
Proportion of elderly in households	0.7076	−0.5008	1.8244***	1.6999**
	(0.4471)	(0.6530)	(0.6394)	(0.7518)
Age of household head	0.0129***	−0.0038	0.0011	−0.0165**
	(0.0047)	(0.0061)	(0.0070)	(0.0079)
Sex of head (male = 1, female = 0)	−0.1260	−0.1689	−0.1347	−0.0613
	(0.1388)	(0.1982)	(0.2004)	(0.2871)
Household head without education degree	Reference			
Household head with primary education	0.3605***	−0.0435	0.1820	−0.0655
	(0.1134)	(0.1436)	(0.1499)	(0.1748)
Household head with lower-secondary	0.4131***	−0.1108	0.5080***	0.3832*
	(0.1247)	(0.1758)	(0.1822)	(0.2207)
Household head with upper-secondary	0.4088***	−0.4701*	0.5343**	0.1113
	(0.1508)	(0.2411)	(0.2201)	(0.3228)
Household head with post-secondary	1.7410***	3.1364***	0.3392	−0.8047
	(0.5241)	(0.6266)	(0.3919)	(0.6589)
Household head without spouse	Reference			
Household head’s spouse without education	−0.2191	0.4239*	0.9196***	0.8790***
	(0.1820)	(0.2436)	(0.2835)	(0.3404)
Household head’s spouse with primary	0.1722	−0.0040	0.4683***	−0.1166
	(0.1154)	(0.1542)	(0.1451)	(0.1822)
Household head’s spouse with lower-	0.0630	−0.0148	0.5328***	−0.5900**
	(0.1322)	(0.1902)	(0.1790)	(0.2376)
Household head’s spouse with upper-	0.4033**	0.2558	0.5491**	−0.1479
	(0.1787)	(0.2996)	(0.2440)	(0.3709)
Household head’s spouse with post-	1.6187**	1.3179	1.4439***	2.4847***
	(0.6789)	(0.8870)	(0.5519)	(0.7544)
Urban (urban = 1; rural = 0)	0.3476***	−0.5764***	0.5644***	−0.0672
	(0.1122)	(0.2011)	(0.1595)	(0.2254)
Number of doctors per 1000 people	2.5881***	2.0884***	−0.1325	−0.1207
	(0.3257)	(0.4800)	(0.4587)	(0.5366)
Number of hospital per 1000 people	−37.7050***	−18.9089	1.5484	52.7175***
	(10.4348)	(15.7174)	(14.1803)	(18.0295)
Dummy year (2008 or 2012)^a^	0.9342***	1.6824***	0.3227**	1.0438***
	(0.0886)	(0.1272)	(0.1270)	(0.1619)
Constant	−8.0450***	9.0468***	−7.8169***	11.5076***
	(0.8438)	(1.2592)	(1.1648)	(1.4951)
Observations	5,013	5,013	4,110	4,110
R-squared	0.188	0.188	0.345	0.345

Note: ^a^Dummy years: for regressions using the 2006 and 2008 VHLSS, the dummy year is equal 1 for 2008 and 0 for 2006. For regressions using the 2010 and 2012 VHLSS, the dummy year is equal 1 for 2012 and 0 for 2010

Robust standard errors in parentheses

Source: Estimates from VHLSSs 2006, 2008, 2010 and 2012

*** *p* < 0.01, ** *p* < 0.05, * *p* < 0.1

In Table 3, we examine the health care outcomes of children over the period 2006–2012. Overall, the health care pattern was quite stable overtime. The percentage of children using health care services was 33.5 in 2006 and 32.8 % in 2012. The number of health care visits decreased slightly from 0.928 in 2006 to 0.854 in 2012. In this study, the health care visits include both outpatient and inpatient health care visits. For children in VHLSSs, only around 3 % of them had inpatient health care visits (around 70 to 80 children in each VHLSS). Thus we cannot examine outpatient and inpatient visits separately.

**Table 3:** Health care variables of children

Health care variables	Children with student health insurance	Children with free health insurance	Children without health insurance	All children
The 2006 VHLSS				
% having health care visits	36.24	38.07	28.42	33.52
Number of health care visits	0.998	1.008	0.806	0.928
Out-of-pocket expenditures per child	113.5	38.3	106.9	103.2
Out-of-pocket expenditures per visit	144.7	53.1	161.4	139.1
The 2008 VHLSS				
% having health care visits	29.49	29.02	23.54	28.34
Number of health care visits	0.726	0.680	0.698	0.713
Out-of-pocket expenditures per child	149.6	71.7	123.1	130.1
Out-of-pocket expenditures per visit	275.6	139.6	219.5	240.9
The 2010 VHLSS				
% having health care visits	36.62	34.33	25.68	34.14
Number of health care visits	1.019	0.869	0.717	0.931
Out-of-pocket expenditures per child	198.1	83.3	102.8	154.8
Out-of-pocket expenditures per visit	315.9	127.7	149.6	250.3
The 2012 VHLSS				
% having health care visits	33.72	31.36	30.67	32.76
Number of health care visits	0.842	0.766	1.112	0.854
Out-of-pocket expenditures per child	169.8	129.0	162.1	158.4
Out-of-pocket expenditures per visit	274.4	280.3	180.9	265.8

Source: Estimates from VHLSSs 2006, 2008, 2010 and 2012

The association between the health insurance programs and the number of health care visits is not clear in Table 3. Insured children had more health care visits than insured children in 2006, but the trend was reverse in 2012. To understand the impact of health insurance, we will use econometric method in the next sections.

### Econometric method

In this study, we will rely on econometric regression methods to estimate the impact of health insurance on health care utilization of children. We start with the basic health capital model of [32, 33], in which the demand for ‘good health’ are inherited stock of health, the shadow price of ‘good health’ commodity, household income and prices of other commodities. The shadow price in turn depends on price of medical care, education, and other factors. Health insurance is expected to decrease the medical care and increase the health care demand.

In our econometric model, we assume a health care variable is a reduced-function of characteristics of households and individuals as follows:

1 $$ {Y}_{it}={\beta}_0+{H}_{it}{\beta}_1+{X}_{it}{\beta}_2+{T}_{it}{\beta}_3+{u}_i+{\varepsilon}_{it} $$

where *Y*_*it*_ is an indicator of health care utilization of child *i* in year *t*. Health care utilization is measured by the number of annual health care visits. *H* is a vector of dummy variables of enrolment in the student health insurance and free health insurance programs (it is equal one for the insured and zero for the uninsured). *X* is a vector of household-level and child-level characteristics. *T* is the time dummy. In the sample of the 2006 and 2008 VHLSSs, *T* equals one for the 2008 year and zero for the 2006 year, and in the sample of the 2010 and 2012 VHLSSs, *T* equals one for the 2012 year and zero for the 2010 year. *u*_*i*_ and *ε*_*it*_ denotes time-invariant and time-variant unobserved variables, respectively. It should be noted that since the number of annual health care visits is a count variable, Poisson regression will be used [34].

The key problem in estimating the impact of health insurance is endogeneity of health insurance. Parent who pay more attention to their children’s health can be more likely to buy health insurance for children and bring their children to health care centers more often when children are sick. In this study, we use panel data fixed-effects regressions to remove the endogeneity bias due to time-invariant unobserved. Fixed-effect regression will, however, fail to remove all endogeneity bias if the unobserved variables which affect health care outcomes and health insurance are not time-invariant.

A source of endogeneity can be adverse selection in health insurance. Adverse selection happens when people with poor health are more likely to enrolled in health insurance [35]. Less healthy children are more likely to have health insurance, and at the same time they are more likely to use health care services. As a result, we can overestimate the impact of health insurance on health care utilization. In our study, the adverse selection might be more likely to occur with the student health insurance program than the free health insurance program, since the student health insurance program is more based on the voluntary bias.

The extent of adverse selection into health insurance has been examined in a large number of studies, and there is not obvious evidence. Several studies find evidence of adverse selection, while other do not find it (e.g., see [36] for a review). In this study, we test the adverse election by running probit regression of children’s enrolment into health insurance in the current period on health variables in the previous. We do not regress health insurance enrolment on health variables in the same period to avoid reverse causality. Having health insurance can improve health by increasing health care utilization. We use the 2006 and 2008 VHLSSs, since these two VHLSSs contain question on whether individuals were sick during the past 4 weeks and the during the past 12 months (before the interview date). Table 7 in Appendix presents the multinomial logit regression of enrolment in health insurance in 2008 on health and health care utilization in 2006 using different model specifications. All the health and health care variables are not significant. The magnitude of point estimate are also very small. Thus we expect that the endogeneity bias in measuring the impact of health insurance is small after child-level and household-level time-invariant unobserved variables and other observed variables are controlled.

Around 65 % of children in Vietnam did not visit health care providers in our data (Table 3). There are not data on out-of-pocket expenditures for children without health care. When there are many zero values of the dependent variable, we can use a Tobit model. However, there are no available fixed-effects Tobit estimators due to an incidental parameter problem in maximum likelihood methods [37]. Instead of fixed-effects Tobit models, we use a two-part model which is often used in health economics to model a variable with a large number of zero values [38, 39]. We estimate the two-part model in the context of fixed-effects panel data as follows:

2 $$ {D}_{it}={\alpha}_D+{H}_{it}{\beta}_D+{X}_{it}{\theta}_D+{T}_t{\gamma}_D+{u}_i+{\varepsilon}_{it}, $$

3 $$ {Y}_{it}={\alpha}_Y+{H}_{it}{\beta}_Y+{X}_{it}{\theta}_Y+{T}_t{\gamma}_Y+{\eta}_i+{v}_{it}\kern0.5em for\kern0.5em {Y}_{it}>0, $$

where *Y*_*it*_ is out-of-pocket expenditures per visit of child *i* in year *t. D*_*it*_ is a binary variable which equals 1 if *Y*_*it*_ > 0 and 0 if *Y*_it_ = 0. *T*_t_ is the time dummy. *H* is health insurance enrolment, and *X* is a vector of other control variables. *u*_*i*_ and *η*_*i*_ denote time-invariant unobserved variables, while *ε*_*it*_ and *v*_*it*_ denote time-variant unobserved variables. Low subscripts *D* and *Y* in parameters of equation (2) and (3) denote parameters in models of *D*_*it*_ and *Y*_*it*_, respectively. We estimate both equations (2) and (3) using fixed-effects regressions.

It should be noted that we estimate equation (2) using a fixed-effects linear probability regression. Linear probability models are also widely used to estimate the effect of an explanatory variable on the probability of the dependent variable (e.g., [40, 41]).^5^ It is easier to interpret the marginal effect from linear probability models.

## Results

Table 4 presents fixed-effects regressions of the number of annual health visits and health expenditures on health insurance. Table 8 in Appendix presents regressions without fixed-effects for comparison. The control variables include log of per capita income, household composition and the number of doctors and the number of hospitals per 1000 people of provinces. The health insurance coverage within a province can be correlated with the capacity of provision of health care services of the province. Variables that time-invariants such as age and gender of children, education and demographic variables of parents are controlled, but they are dropped in the fixed-effects regression. We do not include health status, since these variables are endogenous and might affect health insurance selection. We use a small set of explanatory variables which are exogenous to health insurance. Controlled variables should not be affected by the treatment variable of interest [42, 43].

**Table 4:** Individual fixed-effects regression of health care of children

Explanatory variables	Panel data VHLSSs 2006–2008			Panel data VHLSSs 2010–2012		
	Number of health visits	Having out-of-pocket expenditure on health care	Log of out-of-pocket expenditure per health care visit	Number of health visits	Having out-of-pocket expenditure on health care	Log of out-of-pocket expenditure per health care visit
Student health insurance	0.1242*	0.0077	−0.2732	0.1357*	0.0064	−0.0596
	(0.0676)	(0.0232)	(0.1682)	(0.0770)	(0.0350)	(0.2609)
Free health insurance	0.2008**	0.0144	−0.1580**	0.6605***	0.1398***	−0.6341*
	(0.0948)	(0.0282)	(0.0740)	(0.1178)	(0.0419)	(0.3536)
Enrolment in school	0.0221	0.0249	0.0191	0.0311	0.0212	0.5818
	(0.1129)	(0.0381)	(0.3453)	(0.1392)	(0.0561)	(0.3701)
Log of per capita income	0.0201	0.0234	−0.0077	0.1140	0.0269	−0.1908
	(0.0667)	(0.0273)	(0.1957)	(0.0705)	(0.0300)	(0.1940)
Household size	−0.2693***	−0.0106	−0.1372	0.0116	−0.0105	−0.0141
	(0.0423)	(0.0158)	(0.1435)	(0.0497)	(0.0126)	(0.1406)
Proportion of children	−0.0293	0.0431	1.2379	−0.1327	−0.1829	−0.0789
	(0.2799)	(0.0955)	(0.7870)	(0.3252)	(0.1212)	(0.9634)
Proportion of elderly	0.3749	0.2408	−0.3678	0.2187	0.0948	−0.5288
	(0.6170)	(0.2146)	(1.6220)	(0.7066)	(0.2687)	(1.9081)
Number of doctors per 1000 people	0.4352	−0.0825	2.1253	0.4993	−0.0973	−1.0901
	(0.6049)	(0.1960)	(1.7182)	(0.3444)	(0.1326)	(1.1539)
Number of hospital per 1000 people	−0.1009	−0.0826	−0.0838	0.2649	−0.0772	0.0661
	(0.8291)	(0.0987)	(0.1260)	(0.1944)	(0.0601)	(0.3292)
Dummy year (2008 or 2012)^a^	−0.4648***	−0.0580**	0.4503**	−0.4183***	−0.0472*	0.0191
	(0.0717)	(0.0266)	(0.2210)	(0.0563)	(0.0249)	(0.1675)
Constant		0.2047	3.9243*		0.2988	6.4762***
		(0.2877)	(2.1315)		(0.3188)	(2.1145)
Observations	2,296	5,013	1,279	2,008	4,110	1,094
R-squared		0.005	0.080		0.017	0.033

Note: ^a^Dummy years: for regressions using the 2006 and 2008 VHLSS, the dummy year is equal 1 for 2008 and 0 for 2006. For regressions using the 2010 and 2012 VHLSS, the dummy year is equal 1 for 2012 and 0 for 2010

Robust standard errors in parentheses

Source: Estimates from VHLSSs 2006, 2008, 2010 and 2012

*** *p* < 0.01, ** *p* < 0.05, * *p* < 0.1

It shows that the both school and free health insurance programs have a positive and significant effect on the number of health care visits. The impact of the free health insurance program is higher than the impact of the student health insurance program. Since our model is Possion, our estimate can be interpreted as follows. In the 2006 and 2008 VHLSS sample, the student health insurance and free health insurance programs increased the number of health care visits of children by approximately 12.4 and 20.1 %, respectively. The impact of health insurance was higher in the 2010–2012 period than in the 2006–2008 period. The student health insurance and free health insurance programs increased the number of health care visits of children by approximately 13.6 and 66.1 %, respectively. The finding on the positive effect of health insurance on health care utilization of children is also found in [23] and [29].

The health insurance programs have positive signs in the regressions of the probability of having out-of-pocket health expenditures, but most of them are not significant. Only the effect of the free health insurance on the probability of having out-of-pocket expenditures is significant in the 2010–2012 period. Having free health insurance increased the probability of having out-of-pocket expenditures by 14 %.

Although the point estimates of the student health insurance on out-of-pocket health expenditures per visit are negative, these estimates are not statistically significant. This finding is consistent with findings from [23]. However, the free health insurance reduced the out-of-pocket health expenditures per visit strongly, especially in the period 2010–2012. More specifically, having free health insurance reduced the out-of-pocket health expenditures per visit by around 15.8 in the period 2006–2008 and 63.4 % in the period 2010–2012. This finding implies that health insurance is more useful for the low income households in reducing the burden of health care expenditures.

In Table 5, we include interactions between health insurance and several characteristics of children in regression of health care visits to see whether the effect of health insurance on health care utilization differs for different child groups. Boys and girls have different health status and they can have different patterns of health care utilization. However we do not find a different impact of health insurance between boys and girls.

**Table 5:** Individual fixed-effects regression of the number of health care visits with interactions

Explanatory variables	Panel data VHLSSs 2006–2008				Panel data VHLSSs 2010–2012			
	Model 1	Model 2	Model 3	Model 4	Model 1	Model 2	Model 3	Model 4
Student health insurance	0.0798	0.1624**	-1.1371	-0.2292	0.2800**	0.0067	0.7745	-0.0289
	(0.0898)	(0.0740)	(0.7859)	(0.1965)	(0.1174)	(0.0941)	(1.0848)	(0.1822)
Free health insurance	0.0860	0.0768	-1.7266	0.5155**	0.8661***	0.6073***	3.3580***	-0.1226
	(0.1354)	(0.1019)	(1.3240)	(0.2275)	(0.1815)	(0.1267)	(1.2659)	(0.2643)
Student health insurance × Gender (boy = 1, girl = 0)	0.0965				-0.2511			
	(0.1285)				(0.1543)			
Free health insurance × Gender (boy = 1, girl = 0)	0.2195				-0.3556			
	(0.1821)				(0.2373)			
Student health insurance × Urban		-0.1323				0.3819**		
		(0.1770)				(0.1656)		
Free health insurance × Urban		1.0234***				-0.0708		
		(0.3230)				(0.3996)		
Student health insurance × Log of per capita income			0.1442				-0.0670	
			(0.0892)				(0.1139)	
Free health insurance × Log of per capita income			0.2294				-0.2906**	
			(0.1582)				(0.1348)	
Student health insurance × Number of completed grades of fathers				0.0329				0.0033
				(0.0235)				(0.0220)
Student health insurance × Number of completed grades of mothers				0.4072*				0.2067
				(0.2121)				(0.2157)
Free health insurance × Number of completed grades of fathers				0.0047				0.0845***
				(0.0299)				(0.0316)
Free health insurance × Number of completed grades of mothers				0.3195				0.6301**
				(0.2757)				(0.3156)
Control variables	Yes	Yes	Yes	Yes	Yes	Yes	Yes	Yes
Observations	2,296	2,296	2,296	2,296	2,008	2,008	2,008	2,008
Number of children	1,148	1,148	1,148	1,148	1,004	1,004	1,004	1,004

Note: This Table presents only the coefficients of health insurance and interactions between health insurance and several control variables. Control variables are the same as Table 4

Source: Estimates from VHLSSs 2006, 2008, 2010 and 2012

Robust standard errors in parentheses. *** *p* < 0.01, ** *p* < 0.05, * *p* < 0.1

Some studies find that health care demand is more elastic to the health care cost for the poor households [44, 45]. Because of budget constraints, the poor are less likely to use health care services than the rich. However, health insurance reduces the cost of health care, and as a result the effect of health insurance is expected to be higher on the poor than the rich. Table 5 shows a higher effect of free health insurance for low income households in the period 2010–2012. It implies the important role of health insurance in increasing access to health care services for children in low income households in Vietnam. However, we find a larger effect of health insurance in urban areas than in rural areas, possibly because the high quality of health care services is more available than in urban areas. Urban people are more likely to visit health care providers than rural people when having health insurance.

Finally we include the interactions between parental education (the number of completed educational grades) and health insurance programs. All the interactions have positive sign. The interactions between father’s education and the student health insurance is significant in the 2006–2008 VHLSS panel, while the interactions between mother’s education and the free health insurance is significant in the 2010–2012 VHLSS panel. The impact of health insurance on health care utilization is higher for children with higher education parents. In other words, when having health insurance more educated parents are more likely to use health care services for their children than less educated parents.

## Discussion and Conclusion

Vietnam has been very successful in increasing the coverage of health insurance for the children recently. The percentage of children who were insured with the student health insurance program increased from 52.3 % in 2006 to 62.8 % in 2012. During the same period, the percentage of children insured with the free health insurance program increased from 10.4 % in 2006 to 25.7 % in 2012.

An important question is whether the fast expansion of health insurance is accompanied with an increase in health care utilization for children, especially those in low-income households. This paper examines the effect of student health insurance and free health insurance on the health care utilization and health expenditure for children from 6 to 14 years old in Vietnam using Vietnam Household Living Standard Surveys in 2006, 2008, 2010 and 2012. It finds that both student health insurance and free health insurance programs help children increase the number of health care visits. Health insurance tends to have an increasing impact overtime, and the free health insurance program has a higher impact than student health insurance program. Regarding the impact of out-of-pocket health expenditures per visit, we find that free health insurance but not student health insurance reduces the out-of-pocket health expenditures per visit strongly, especially in the period 2010–2012. The effect of health insurance on health utilization is higher for children in low-income households and high-education parents.

This study shows the important role of health insurance in accessing health care services for children, especially those from for low income households in Vietnam. Accordingly, provision of health insurance can contribute to improve access to health care services for children. This study also highlights the importance role of education of parents in children’s health care. Children with more educated parents are more likely not only to have health insurance but also to use it. Thus, improving knowledge and education for parents is important in increasing the coverage of health insurance as well as health care utilization for children.

## Appendix

**Table 6:** Descriptive statistics of explanatory variables

Variables	Type	VHLSS 2006	VHLSS 2008	VHLSS 2010	VHLSS 2012
Age of child	Discrete	9.975	11.945	9.576	11.411
		(0.045)	(0.046)	(0.052)	(0.053)
Sex (male = 1, female = 0)	Binary	0.502	0.502	0.524	0.524
		(0.010)	(0.010)	(0.011)	(0.011)
Ethnic minorities (ethnic minorities = 1, Kinh/Hoa = 0)	Binary	0.154	0.154	0.204	0.207
		(0.007)	(0.007)	(0.009)	(0.009)
Attending school (yes = 1, no = 0)	Binary	0.953	0.907	0.957	0.929
		(0.004)	(0.006)	(0.004)	(0.006)
Log of per capita income	Continuous	8.570	8.868	9.266	9.675
		(0.013)	(0.014)	(0.017)	(0.016)
Household size	Discrete	5.112	5.019	4.860	4.806
		(0.032)	(0.032)	(0.033)	(0.030)
Proportion of children in households	Continuous	0.424	0.364	0.430	0.390
		(0.003)	(0.003)	(0.003)	(0.004)
Proportion of elderly in households	Continuous	0.047	0.048	0.045	0.047
		(0.002)	(0.002)	(0.002)	(0.002)
Age of household head	Discrete	43.713	45.020	42.537	43.989
		(0.238)	(0.224)	(0.252)	(0.244)
Sex of head (male = 1, female = 0)	Binary	0.805	0.803	0.822	0.817
		(0.008)	(0.008)	(0.008)	(0.009)
Household head without education degree	Binary	0.259	0.238	0.272	0.254
		(0.009)	(0.009)	(0.010)	(0.010)
Household head with primary education	Binary	0.280	0.292	0.293	0.272
		(0.009)	(0.009)	(0.010)	(0.010)
Household head with lower-secondary	Binary	0.269	0.274	0.220	0.254
		(0.009)	(0.009)	(0.009)	(0.010)
Household head with upper-secondary	Binary	0.164	0.166	0.159	0.160
		(0.007)	(0.007)	(0.008)	(0.008)
Household head with post-secondary	Binary	0.027	0.030	0.058	0.059
		(0.003)	(0.003)	(0.005)	(0.005)
Household head without spouse	Binary	0.136	0.141	0.120	0.122
		(0.007)	(0.007)	(0.007)	(0.007)
Household head’s spouse without education degree	Binary	0.225	0.213	0.261	0.253
		(0.008)	(0.008)	(0.010)	(0.010)
Household head’s spouse with primary education	Binary	0.270	0.281	0.275	0.263
		(0.009)	(0.009)	(0.010)	(0.010)
Household head’s spouse with lower-secondary	Binary	0.222	0.226	0.198	0.219
		(0.008)	(0.008)	(0.009)	(0.009)
Household head’s spouse with upper-secondary	Binary	0.123	0.112	0.101	0.098
		(0.007)	(0.006)	(0.007)	(0.007)
Household head’s spouse with post-secondary	Binary	0.024	0.028	0.046	0.045
		(0.003)	(0.003)	(0.005)	(0.005)
Urban (urban = 1; rural = 0)	Binary	1.788	1.788	0.223	0.225
		(0.008)	(0.008)	(0.009)	(0.009)
Number of doctors per 1000 people	Continuous	0.499	0.424	0.555	0.565
		(0.003)	(0.002)	(0.004)	(0.004)
Number of hospital per 1000 people	Continuous	0.010	0.009	0.016	0.019
		(0.000)	(0.000)	(0.000)	(0.000)
Number of observations		2507	2507	2055	2055

Standard errors in parentheses. Source: Estimates from VHLSSs 2006, 2008, 2010 and 2012

**Table 7:** Multinomial logit regression of enrolment in health insurance in 2008 on the 2006 explanatory variables

Explanatory variables	Model 1		Model 2		Model 3	
	Enrolled in student health insurance in 2008 (yes = 1, no = 0)	Enrolled in free health insurance in 2008 (yes = 1, no = 0)	Enrolled in student health insurance in 2008 (yes = 1, no = 0)	Enrolled in free health insurance in 2008 (yes = 1, no = 0)	Enrolled in student health insurance in 2008 (yes = 1, no = 0)	Enrolled in free health insurance in 2008 (yes = 1, no = 0)
Being sick in the past 4 weeks	0.3113	0.3938	0.3281	0.3710	0.3199	0.4179
	(0.2432)	(0.2726)	(0.2355)	(0.2291)	(0.1982)	(0.2606)
Being sick in the past 12 months	0.1221	0.1055	0.1075	0.0760	0.0898	0.0613
	(0.0915)	(0.1572)	(0.1052)	(0.1503)	(0.1062)	(0.1387)
Sex (male = 1, female = 0)	0.0752	−0.0018	0.0532	−0.0172	0.0464	−0.0880
	(0.1122)	(0.1117)	(0.1188)	(0.1178)	(0.1224)	(0.1128)
Age of child	0.0070	−0.0705***	−0.0128	−0.0859***	−0.0121	−0.0850***
	(0.0168)	(0.0236)	(0.0191)	(0.0257)	(0.0188)	(0.0299)
Ethnic minorities	0.2941	1.4191***	0.5921**	1.6516***	0.8573***	1.1182***
	(0.2171)	(0.3302)	(0.2532)	(0.3670)	(0.2716)	(0.4275)
Urban	0.3138	−0.7767***	0.1799	−0.8826***	−0.0960	−0.8965**
	(0.1954)	(0.2832)	(0.1913)	(0.2642)	(0.1810)	(0.3613)
Having health insurance in 2006			0.9595***	0.7294***	0.9028***	0.7468***
			(0.1742)	(0.2230)	(0.1696)	(0.2248)
Number of health care visit in 2006			−0.0046	0.0084	0.0005	0.0262
			(0.0306)	(0.0308)	(0.0308)	(0.0308)
Out-of-pocket expenditures on health in 2006 (million VND)			−0.0665**	−0.0186	−0.0773*	−0.0467
			(0.0337)	(0.0618)	(0.0397)	(0.0477)
Log of per capita income					0.1905	−1.4348***
					(0.1450)	(0.1629)
Age of household head					0.0049	0.0008
					(0.0044)	(0.0069)
Sex of head (male = 1, female = 0)					0.0300	−0.0985
					(0.2121)	(0.2962)
Household head without education degree	Reference					
Household head with primary education					0.5921***	0.3111
					(0.1578)	(0.2640)
Household head with lower-secondary					0.6522***	0.1530
					(0.2353)	(0.2218)
Household head with upper-secondary					0.8411***	0.3907
					(0.2596)	(0.4023)
Household head with post-secondary					2.0384***	2.9468***
					(0.6885)	(0.9658)
Household head without spouse	Reference					
Household head’s spouse without education degree					−0.1766	0.5689*
					(0.2725)	(0.3336)
Household head’s spouse with primary education					−0.1890	−0.0817
					(0.1879)	(0.3232)
Household head’s spouse with lower-secondary					−0.0897	0.2781
					(0.2039)	(0.2676)
Household head’s spouse with upper-secondary					−0.0109	0.2500
					(0.3387)	(0.3738)
Household head’s spouse with post-secondary					0.6977	0.6813
					(0.7832)	(1.1070)
Constant	0.8948***	0.5061	0.6043**	0.3018	−1.7134	12.5378***
	(0.3041)	(0.3635)	(0.2986)	(0.3654)	(1.2174)	(1.5429)
Observations	2,486	2,486	2,486	2,486	2,486	2,486
Pseudo R-squared	0.0471	0.0471	0.0625	0.0625	0.129	0.129

Robust standard errors in parentheses

Source: Estimates from VHLSSs 2006, 2008, 2010 and 2012

*** *p* < 0.01, ** *p* < 0.05, * *p* < 0.1

**Table 8:** Regressions of health care of children

Explanatory variables	Panel data VHLSSs 2006–2008			Panel data VHLSSs 2010–2012		
	Poisson regression: Number of health visits	OLS: Having out-of-pocket expenditure on health care	OLS: Log of out-of-pocket expenditure per health care visit	Poisson regression: Number of health visits	OLS: Having out-of-pocket expenditure on health care	OLS: Log of out-of-pocket expenditure per health care visit
Student health insurance	0.0982	0.0150	−0.2119**	0.1013	0.0222	0.0609
	(0.0950)	(0.0160)	(0.1008)	(0.1247)	(0.0220)	(0.1202)
Free health insurance	0.3608***	0.0491**	−0.5050***	0.1437	0.0299	0.0212
	(0.1169)	(0.0217)	(0.1243)	(0.1378)	(0.0246)	(0.1440)
Enrolment in school	−0.1301	0.0037	−0.0778	−0.3683**	−0.0335	0.1840
	(0.1518)	(0.0242)	(0.2003)	(0.1842)	(0.0306)	(0.1839)
Log of per capita income	0.2186***	0.0622***	0.1773***	0.0842	0.0538***	0.3878***
	(0.0524)	(0.0117)	(0.0643)	(0.0614)	(0.0127)	(0.0762)
Household size	−0.1585***	−0.0194***	−0.0098	−0.1322***	−0.0319***	−0.0129
	(0.0260)	(0.0044)	(0.0280)	(0.0319)	(0.0051)	(0.0331)
Proportion of children	0.0783	0.1296***	0.1677	0.5256**	−0.0290	0.2029
	(0.2461)	(0.0434)	(0.2932)	(0.2648)	(0.0498)	(0.3386)
Proportion of elderly	0.1184	0.0358	−0.5145	0.5754	0.0128	0.1305
	(0.3008)	(0.0676)	(0.3766)	(0.3584)	(0.0759)	(0.4110)
Number of doctors per 1000 people	−0.5454*	−0.0780	0.1115	−1.3702***	−0.2046***	1.5473***
	(0.3140)	(0.0580)	(0.3025)	(0.3341)	(0.0639)	(0.4143)
Number of hospital per 1000 people	−0.3128**	−0.0716***	0.9451	0.1576	0.0346	−0.4301***
	(0.1284)	(0.0164)	(1.2841)	(0.0962)	(0.0214)	(0.1280)
Dummy year (2008 or 2012)	−0.6285***	−0.0654***	0.4575***	−0.3741***	−0.0769***	−0.1087
	(0.0782)	(0.0146)	(0.0867)	(0.0830)	(0.0167)	(0.0954)
Constant	−0.5512	−0.1144	2.2843***	0.3076	0.0346	0.4203
	(0.5732)	(0.1163)	(0.6500)	(0.6882)	(0.1312)	(0.8111)
Observations	5,013	5,013	1,279	4,110	4,110	1,094
R-squared		0.027	0.052		0.028	0.057

Note: Dummy years: for regressions using the 2006 and 2008 VHLSS, the dummy year is equal 1 for 2008 and 0 for 2006. For regressions using the 2010 and 2012 VHLSS, the dummy year is equal 1 for 2012 and 0 for 2010

Robust standard errors in parentheses

Source: Estimates from VHLSSs 2006, 2008, 2010 and 2012

*** *p* < 0.01, ** *p* < 0.05, * *p* < 0.1

## Footnotes

1 See [36] and [46] for review of empirical studies on the impact of health insurance.

2 Vietnam is divided into 63 provinces. Each province is divided into districts and each district is further divided into communes. Communes are smallest administrative divisions in Vietnam. In 2009, there are 684 districts and 11,112 communes (according to the Population and Housing Census 2009).

3 Formal sector include workers enrolled in social insurance.

4 They are both based on the voluntary and payment basis.

5 Another model that can be used to model the dependent variable with zero values is the hurdle binomial model. The first stage of the hurdle binomial model is to use the probit model to estimate the probability of the dependent variable having positive values. The second stage of the hurdle binomial model is to use a Poisson model to model the dependent variable using the sample with positive values of the dependent variable. In this paper, we do not use the hurdle binomial model, since we used fixed-effects linear probability model in the first stage.
